# Mitochondria as new therapeutic targets for eradicating cancer stem cells: Quantitative proteomics and functional validation via MCT1/2 inhibition

**DOI:** 10.18632/oncotarget.2789

**Published:** 2014-11-15

**Authors:** Rebecca Lamb, Hannah Harrison, James Hulit, Duncan L. Smith, Michael P. Lisanti, Federica Sotgia

**Affiliations:** ^1^ The Manchester Centre for Cellular Metabolism (MCCM), Institute of Cancer Sciences, University of Manchester; ^2^ The Breakthrough Breast Cancer Research Unit, Institute of Cancer Sciences, University of Manchester; ^3^ The Cancer Research UK Manchester Institute, University of Manchester

**Keywords:** mitochondrial markers, cancer stem cells, proteomic analysis, ketone metabolism, monocarboxylate transporters (MCTs), AR-C155858, AZD3965, CHCHD2

## Abstract

Here, we used quantitative proteomics analysis to identify novel therapeutic targets in cancer stem cells and/or progenitor cells. For this purpose, mammospheres from two ER-positive breast cancer cell lines (MCF7 and T47D) were grown in suspension using low-attachment plates and directly compared to attached monolayer cells grown in parallel. This allowed us to identify a subset of proteins that were selectively over-expressed in mammospheres, relative to epithelial monolayers. We focused on mitochondrial proteins, as they appeared to be highly upregulated in both MCF7 and T47D mammospheres. Key mitochondrial-related enzymes involved in beta-oxidation and ketone metabolism were significantly upregulated in mammospheres, as well as proteins involved in mitochondrial biogenesis, and specific protein inhibitors of autophagy/mitophagy. Overall, we identified >40 “metabolic targets” that were commonly upregulated in both MCF7 and T47D mammospheres. Most of these “metabolic targets” were also transcriptionally upregulated in human breast cancer cells *in vivo*, validating their clinical relevance. Based on this analysis, we propose that increased mitochondrial biogenesis and decreased mitochondrial degradation could provide a novel mechanism for the accumulation of mitochondrial mass in cancer stem cells. To functionally validate our observations, we utilized a specific MCT1/2 inhibitor (AR-C155858), which blocks the cellular uptake of two types of mitochondrial fuels, namely ketone bodies and L-lactate. Our results indicate that inhibition of MCT1/2 function effectively reduces mammosphere formation, with an IC-50 of ~1 μM, in both ER-positive and ER-negative breast cancer cell lines. Very similar results were obtained with oligomycin A, an inhibitor of the mitochondrial ATP synthase. Thus, the proliferative clonal expansion of cancer stem cells appears to require oxidative mitochondrial metabolism, related to the re-use of monocarboxylic acids, such as ketones or L-lactate. Our findings have important clinical implications for exploiting mitochondrial metabolism to eradicate cancer stem cells and to prevent recurrence, metastasis and drug resistance in cancer patients. Importantly, a related MCT1/2 inhibitor (AZD3965) is currently in phase I clinical trials in patients with advanced cancers: http://clinicaltrials.gov/show/NCT01791595.

## INTRODUCTION

Cancer stem cells (CSCs), or tumor-initiating cells (TICs), are thought to be resistant to conventional anti-cancer therapies, and have been implicated in treatment failure, tumor recurrence and distant metastasis [1, 2]. Thus, residual treatment-resistant cancer stem cells are believed to be responsible for poor clinical outcomes in most cancer types [2-4]. Since CSCs are relatively rare and elusive, very little is known about them, especially regarding their physiology and metabolic phenotype.

Consistent with the idea that CSCs are resistant to cellular stress, they are able to undergo anchorage-independent growth in low-attachment plates, allowing the formation of 3D spheroids with the properties of cancer stem cells and/or progenitor cells [5]. Under these suspension conditions, most epithelial cancer cells undergo a specialized form of cell death/apoptosis, termed anoikis. Importantly, each of these 3D spheroids is formed from the anchorage-independent clonal expansion of a single cancer stem cell, and not from the self-aggregation of existing cancer cells [5]. Therefore, the preparation of 3D spheroid cultures provides a functional assay to enrich for a population of cells with an epithelial stem cell-like phenotype. In this regard, the behavior of 3D spheroids (also known as mammosphere cultures) prepared from primary breast cancer cells or breast cancer epithelial cell lines are the most well-characterized.

Here, we isolated large numbers of mammospheres from two independent ER-positive breast cancer cell lines, namely MCF7 and T47D cells, in an attempt to better understand their phenotypic behavior at a molecular level. The large-scale preparation of mammospheres allowed us to then perform unbiased label-free proteomics analysis, in an attempt to understand the proteome that is characteristic of cancer stem cells. Interestingly, based on this initial analysis, we noticed that mammospheres dramatically overexpress mitochondrial-related proteins. Thus, we focused on the mitochondrial proteins that were upregulated, relative to cells cultured as epithelial monolayers, in parallel. Based on this analysis, we speculate that CSCs become resistant to stress by fortifying their capacity to produce ATP by oxidative mitochondrial metabolism. Treatment with the MCT1/2 inhibitor (AR-C155858) validated that mammosphere formation is dependent on the uptake of specific mitochondrial fuels, such as L-lactate and ketone bodies.

Importantly, over the last several decades, significant progress has been made in understanding the critical role of cellular metabolism in tumor initiation, progression and metastasis, including studies related to ROS production, oxidative stress, glycolysis, glutamine metabolism and oxidative mitochondrial metabolism [6-13]. However, most of these studies have focused on “bulk” cancer cells, but not on cancer stem cells or the progenitor cell population. Thus, there is a great need to understand tumor metabolism in the specific context of “stemness”, to identify a metabolic “Achilles' Heel” to eradicate cancer stem cells. As such, our current findings provide an unbiased systematic approach for identifying new metabolic targets in cancer stem cells, using quantitative proteomics analysis, and a strong rationale for therapeutically targeting L-lactate, ketone bodies and mitochondrial metabolism in the cancer stem cell population.

## RESULTS

### Proteomic analysis of MCF7 mammospheres

Monolayer and mammosphere cultures of MCF7 cells, an ER-positive cell line, were subjected to quantitative label-free proteomics analysis. Greater than 500 proteins were found to be upregulated or downregulated. For simplicity, we focused on the proteins that were significantly upregulated in mammospheres, relative to mononlayer cell cultures (p < 0.05). Immediately, we noticed that several mitochondrial proteins were highly upregulated in mammospheres, so we restricted our analysis to mitochondrial proteins and key related metabolic enzymes.

Table 1 shows a non-redundant list of 62 mitochondrial-related proteins that were selectively upregulated in MCF7 mammospheres. Only proteins with a fold increase of ~2 or greater were selected for this analysis. Note that 9 mitochondrial proteins were infinitely upregulated, as compared with monolayer cultures. A functional analysis of this list revealed that 12 proteins were specifically related to beta-oxidation and ketone metabolism/re-utilization (HSD17B10, BDH1, ACAT1, ACADVL, ACACA, ACLY, HADHB, SUCLG2, ACAD9, HADHA, ECHS1, ACADSB). Also, 8 proteins involved in mitochondrial biogenesis were dramatically upregulated (HSPA9, TIMM8A, GFM1, MRPL45, MRPL17, HSPD1(HSP60), TSFM, TUFM). In addition, many proteins related to electron transport (NDUFB10, COX6B1, PMPCA, COX5B, SDHA, UQCRC1), ATP synthesis (ATP5B, ATPIF1, ATP5A1, ATP5F1, ATP5H, ATP5O), ADP/ATP exchange/transport (SLC25A5), CoQ synthesis (COQ9), or ROS production (GPD2) were also increased. Finally, two proteins involved in the suppression of glycolysis, autophagy and mitophagy were also significantly increased (SOGA1, LRPPRC). Thus, increased mitochondrial biogenesis and decreased mitochondrial degradation could provide a novel mechanism for the overall accumulation of mitochondrial mass in cancer stem cells.

**Table 1:** Mitochondrial-related Proteins Up-regulated in MCF7 Mammospheres

Symbol	Gene Description	Fold-Upregulation	ANOVA
AK2	Adenylate kinase 2, mitochondrial	Infinity	7.43E-14
ATP5B	ATP synthase subunit beta	Infinity	3.80E-08
GPD2	Glycerol-3-phosphate dehydrogenase, mitochondrial	Infinity	7.80E-13
SOGA1	Suppressor of glycolysis and autophagy 1	Infinity	5.59E-13
CHCHD2	Coiled-coil-helix-coiled-coil-helix domain-containing protein 2, mitochondrial	Infinity	<1.0E-17
CPOX	Coproporphyrinogen-III oxidase, mitochondrial	Infinity	6.70E-12
**HADH2**	Hydroxysteroid (17-Beta) Dehydrogenase 10; HSD17B10 protein	Infinity	1.86E-13
MCCC1	Methylcrotonoyl-CoA carboxylase subunit alpha, mitochondrial	Infinity	2.80E-12
SLC25A10	Mitochondrial dicarboxylate carrier	Infinity	5.82E-05
HSPA9	Stress-70 protein, mitochondrial	298,325.4	2.62E-13
TIMM8A	Mitochondrial import inner membrane translocase subunit Tim8 A	36,902.6	1.45E-11
**BDH1**	D-beta-hydroxybutyrate dehydrogenase, mitochondrial	2,592.8	1.23E-10
**ACAT1**	Acetyl-CoA acetyltransferase, mitochondrial	1,124.9	1.82E-07
NDUFB10	NADH dehydrogenase [ubiquinone] 1 beta subcomplex subunit 10	975.80	2.22E-09
COX6B1	Cytochrome c oxidase subunit 6B1	622.58	3.86E-05
**ACADVL**	Very-long-chain specific acyl-CoA dehydrogenase, mitochondrial	573.07	3.23E-06
DHTKD1	2-oxoglutarate dehydrogenase E1 component DHKTD1, mitochondrial	355.26	1.11E-07
CCDC47	Coiled-coil domain-containing protein 47	328.85	3.15E-10
PGD	6-phosphogluconate dehydrogenase (pentose phosphate shunt)	292.09	4.14E-06
**ACACA**	Acetyl-Coenzyme A carboxylase alpha	224.71	1.40E-09
PC	Pyruvate carboxylase, mitochondrial	158.10	9.09E-05
VDAC3	Voltage-dependent anion-selective channel protein 3	136.20	2.97E-08
ALDH4A1	Delta-1-pyrroline-5-carboxylate dehydrogenase, mitochondrial	131.96	3.93E-05
ECH1	Delta(3,5)-Delta(2,4)-dienoyl-CoA isomerase, mitochondrial	114.95	3.27E-05
**ACLY**	ATP Citrate Lyase, cytosolic	100.67	7.99E-08
GFM1	Mitochondrial elongation factor G	97.22	1.32E-05
PMPCA	Mitochondrial-processing peptidase alpha subunit; paralog is UQCRC2	79.00	2.57E-09
**HADHB**	Mitochondrial trifunctional protein beta subunit	60.00	7.71E-09
NNT	NAD(P) transhydrogenase, mitochondrial	50.38	4.20E-10
MRPL45	39S ribosomal protein L45, mitochondrial	46.42	4.32E-11
**SUCLG2**	Succinyl-CoA ligase [GDP-forming] subunit beta, mitochondrial	32.18	6.24E-09
LRPPRC	Leucine-rich PPR motif-containing protein, mitochondrial	30.92	2.63E-12
DLST	Dihydrolipoyllysine succinyltransferase, 2-oxoglutarate dehydrogenase	23.99	2.69E-08
DLAT	Dihydrolipoyllysine acetyltransferase, pyruvate dehydrogenase complex	23.94	5.07E-12
HSPD1	60 kDa heat shock protein, mitochondrial	16.44	0.0001
**ACAD9**	Acyl-CoA dehydrogenase family member 9, mitochondrial	15.51	1.90E-10
PTCD3	Pentatricopeptide repeat-containing protein 3, mitochondrial	14.10	4.17E-05
HARS2	Histidine--tRNA ligase, mitochondrial	13.05	2.30E-08
SDHA	Succinate dehydrogenase (ubiquinone) flavoprotein subunit, mitochondrial	10.62	3.74E-11
ATPIF1	ATPase inhibitor, mitochondrial	10.13	0.025
CKMT1/2	Creatine kinase, ubiquitous mitochondrial (EC 2.7.3.2)	8.32	4.37E-10
ACO2	Aconitate hydratase, mitochondrial (EC 4.2.1.3)	8.09	4.79E-11
COX5B	Cytochrome c oxidase subunit 5B, mitochondrial	7.69	6.50E-09
MCCC2	Methylcrotonoyl-CoA carboxylase beta chain, mitochondrial	7.54	2.28E-07
CKMT1B	Creatine kinase U-type, mitochondrial	6.45	3.51E-06
SLC25A1	Tricarboxylate transport protein, mitochondrial	5.71	1.22E-05
MRPL17	39S ribosomal protein L17, mitochondrial	4.80	5.31E-05
**HADHA**	Trifunctional enzyme subunit alpha, mitochondrial	4.70	1.91E-10
**ECHS1**	Enoyl-CoA hydratase, mitochondrial	4.50	3.33E-07
LETM1	LETM1 and EF-hand domain-containing protein 1, mitochondrial	4.16	2.24E-11
TSFM	Elongation factor Ts, mitochondrial	3.75	0.002
UQCRC1	Cytochrome b-c1 complex subunit 1, mitochondrial	3.14	0.002
ATP5A1	ATP synthase subunit alpha, mitochondrial	3.05	0.001
PPA2	Inorganic pyrophosphatase 2, mitochondrial	2.78	0.002
COQ9	Ubiquinone biosynthesis protein COQ9, mitochondrial	2.69	0.001
ATP5F1	ATP synthase, H+ transporting, mitochondrial F0 complex, subunit B1	2.51	0.0005
SLC25A5	Solute carrier family 25 (adenine nucleotide translocator), member 5	2.46	0.002
TUFM	Elongation factor Tu, mitochondrial	2.31	0.0007
KIAA0664	Clustered mitochondria protein homolog	2.31	0.0001
ATP5H	ATP synthase subunit d, mitochondrial	2.27	6.20E-05
**ACADSB**	Short/branched chain specific acyl-CoA dehydrogenase, mitochondrial	2.10	0.004
ATP5O	ATP synthase subunit O, mitochondrial	1.92	0.0002

**Enzymes in BOLD are related to beta-oxidation and ketone metabolism.**

### Proteomic analysis of T47D mammospheres

For comparison purposes, we also performed unbiased label-free proteomic analysis on a second independent ER-positive breast cancer cell line, namely T47D cells.

**Figure 1:**
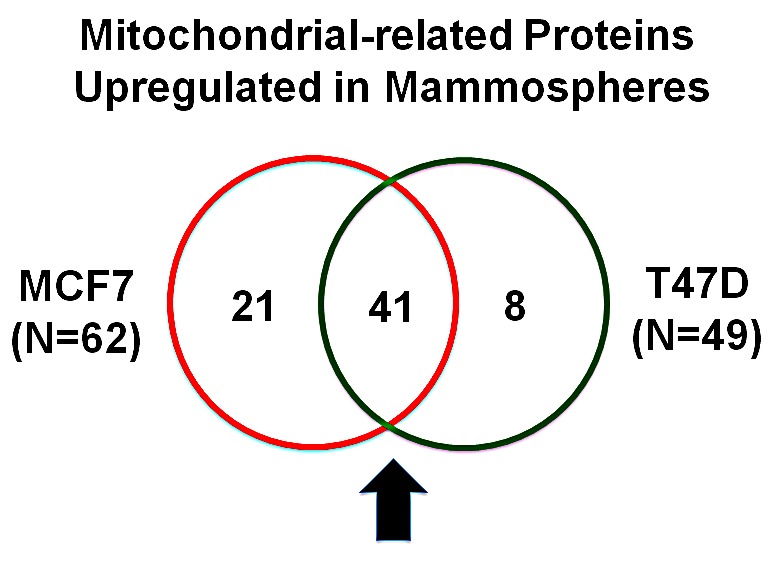
Venn diagram highlighting the conserved upregulation of mitochondrial related proteins in both MCF7 and T47D mammospheres Note that >40 mitochondrial-related proteins were commonly upregulated in both data sets.

Our results are summarized in Table 2. Note that 49 mitochondrial-related proteins were specifically over-expressed in T47D mammospheres, as compared with T47D monolayer cultures processed in parallel. Remarkably, 41 of these proteins overlapped with the proteins that were upregulated in MCF7 mammospheres (41/49 = 84% overlap). See the Venn diagram presented in Figure 1. Therefore, many of the same biological processes would be expected to be activated or enhanced. Thus, beta-oxidation, ketone re-utilization, mitochondrial biogenesis, and ROS production, with decreased mitochondrial degradation and reduced autophagy, are likely to be key biological features of both MCF7 and T47D mammospheres. Interestingly, CHCHD2 and CPOX were infinitely upregulated in both MCF7 and T47D data sets.

**Table 2:** Mitochondrial-related Proteins Up-regulated in T47D Mammospheres

Symbol	Gene Description	Fold-Upregulation	ANOVA
**CHCHD2**	Coiled-coil-helix-coiled-coil-helix domain-containing protein 2, mitochondrial	Infinity	<1.0E-17
**CPOX**	Coproporphyrinogen-III oxidase, mitochondrial	Infinity	<1.0E-17
**HSPD1**	60 kDa heat shock protein, mitochondrial	69.06	1.45E-05
**ACADVL**	Very-long-chain specific acyl-CoAdehydrogenase, mitochondrial	66.62	2.57E-06
**TIMM8A**	Mitochondrial import inner membrane translocase subunit Tim8 A	50.35	2.45E-06
**ACAT1**	Acetyl-CoA acetyltransferase, mitochondrial	49.45	4.40E-10
**HADH2**	Hydroxysteroid (17-Beta) Dehydrogenase 10; HSD17B10 protein	47.72	3.47E-05
**HADHB**	Mitochondrial trifunctional protein beta subunit	37.42	2.40E-12
**ACACA**	Acetyl-Coenzyme A carboxylase alpha	34.85	7.27E-11
**PGD**	6-phosphogluconate dehydrogenase (pentose phosphate shunt)	23.93	0.03
**DLST**	Component of 2-oxoglutarate dehydrogenase complex, mitochondrial	21.39	3.19E-14
PDHB	Pyruvate dehydrogenase E1 component subunit beta, mitochondrial	19.84	0.01
**MCCC1**	Methylcrotonoyl-CoA carboxylase subunit alpha, mitochondrial	18.34	0.01
**DHTKD1**	2-oxoglutarate dehydrogenase E1 component DHKTD1, mitochondrial	17.54	0.0006
**ATPIF1**	ATPase inhibitor, mitochondrial	16.36	0.01
**PMPCA**	Mitochondrial-processing peptidase alpha subunit; paralog of UQCRC2	15.96	1.83E-13
**BDH1**	D-beta-hydroxybutyrate dehydrogenase, mitochondrial	15.17	0.0003
**SLC25A10**	Mitochondrial dicarboxylate carrier	13.81	0.015
**VDAC3**	Voltage-dependent anion-selective channel protein 3	12.91	1.11E-08
MRPL47	39S ribosomal protein L47, mitochondrial	10.69	0.03
**ECH1**	Delta(3,5)-Delta(2,4)-dienoyl-CoA isomerase, mitochondrial	10.49	8.18E-08
**MCCC2**	Methylcrotonoyl-CoA carboxylase beta chain, mitochondrial	10.38	1.52E-06
**NNT**	NAD(P) transhydrogenase, mitochondrial	10.08	4.46E-10
**ALDH4A1**	Delta-1-pyrroline-5-carboxylate dehydrogenase, mitochondrial	8.18	9.73E-05
**LRPPRC**	Leucine-rich PPR motif-containing protein, mitochondrial	8.17	6.62E-10
**SUCLG2**	Succinyl-CoA ligase [GDP-forming] subunit beta, mitochondrial	7.54	3.10E-06
**ACLY**	ATP Citrate Lyase, cytosolic	7.01	0.01
**CKMT1/2**	Creatine kinase, ubiquitous mitochondrial	6.81	4.73E-11
**MRPL45**	39S ribosomal protein L45, mitochondrial	6.02	9.00E-11
**GPD2**	Glycerol-3-phosphate dehydrogenase, mitochondrial	5.99	0.002
C21orf33	ES1 protein homolog, mitochondrial	5.97	0.005
**HARS2**	Probable histidine--tRNA ligase, mitochondrial	5.82	2.03E-07
**PTCD3**	Pentatricopeptide repeat-containing protein 3, mitochondrial	5.57	0.01
SQRDL	Sulfide:quinone oxidoreductase, mitochondrial	4.94	1.15E-08
**ATP5F1**	ATP synthase, H+ transporting, mitochondrial F0 complex, subunit B1	4.38	0.0005
**DLAT**	Dihydrolipoyllysine acetyltransferase, pyruvate dehydrogenase complex	4.31	6.71E-09
**HSPA9**	Stress-70 protein, mitochondrial	4.14	0.03
**LETM1**	LETM1 and EF-hand domain-containing protein 1, mitochondrial	4.14	0.01
**PC**	Pyruvate carboxylase, mitochondrial	3.39	1.33E-05
**MRPL17**	39S ribosomal protein L17, mitochondrial	3.30	0.01
**SLC25A1**	Tricarboxylate transport protein, mitochondrial	3.22	0.03
PDCD8	Apoptosis-inducing factor 1, mitochondrial	3.22	5.92E-06
**UQCRC1**	Cytochrome b-c1 complex subunit 1, mitochondrial	2.94	1.16E-05
**SOGA1**	Suppressor of glycolysis and autophagy 1	2.90	1.01E-05
**CKMT1A**	Creatine kinase U-type, mitochondrial	2.90	5.42E-05
**CKMT1B**	Creatine kinase U-type, mitochondrial	2.66	5.43E-05
CPT1A	Carnitine palmitoyltransferase 1A, mitochondrial protein	2.51	0.0005
ACADS	Medium-chain specific acyl-CoA dehydrogenase, mitochondrial (EC 1.3.99.3)	2.47	0.0001
MRPS22	28S ribosomal protein S22, mitochondrial	2.12	0.0001

Proteins shown in BOLD were also up-regulated in MCF7 Mammospheres.

### Functional effects of MCT1/2 inhibition on mammosphere formation

Next, to functionally validate the hypothesis that mammosphere formation may require ketone re-utilization and oxidative mitochondrial metabolism, we used a highly specific inhibitor (AR-C155858) of the relevant monocarboxylate transporters, namely MCT1/2 [14, 15]. MCT1/2 normally function as specific transporters for the uptake of ketone and L-lactate [16]. AR-C155858 effectively inhibits MCT1/2 function, and blocks the cellular uptake of both ketone bodies and L-lactate [14, 15].

**Figure 2:**
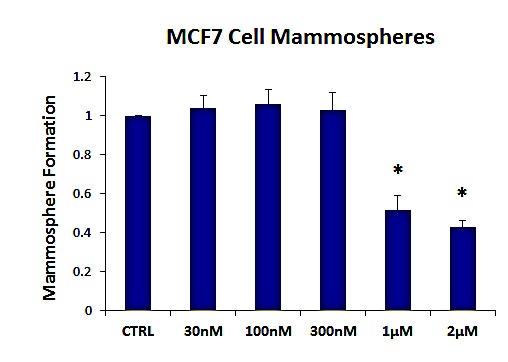
The MCT1/2 inhibitor AR-C155858 significantly reduces mammosphere formation in MCF7 cells Increasing concentrations of AR-C155858 inhibit mammosphere formation, using an ER-positive cell line (MCF7). Importantly, AR-C155858 significantly reduces mammosphere formation, with an IC-50 of ~ 1 μM. The vehicle-alone control was normalized to one. (*)p <6.0E-06.

Figure 2 shows the effects of increasing concentrations of AR-C155858 on mammosphere formation, using an ER-positive cell line (MCF7). Importantly, the MCT1/2 inhibitor AR-C155858 significantly reduces mammosphere formation, with an IC-50 of ~ 1μM.

**Figure 3:**
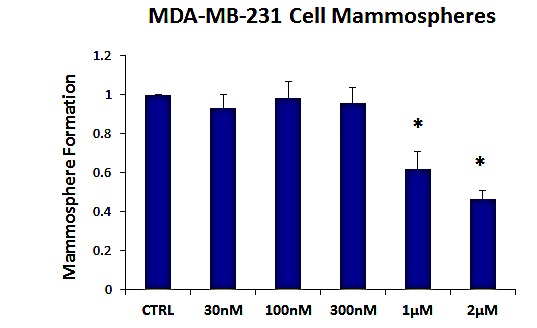
The MCT1/2 inhibitor AR-C155858 significantly reduces mammosphere formation in MDA-MB-231 cells Note that AR-C155858 also effectively reduces mammosphere formation in this cellular context, with an IC-50 of ~ 1-2 μM. The vehicle-alone control was normalized to one. (*)p <0.0005.

As breast cancer stem cells are thought to be ER-negative, we also evaluated the effects of AR-C155858 on an ER-negative cell line, namely MDA-MB-231 cells. Figure 3 shows that the MCT1/2 inhibitor AR-C155858 also effectively reduces mammosphere formation in this cellular context, with an IC-50 of ~ 1-2 μM. Therefore, MCT1/2 inhibition may be a new general therapeutic strategy that could be utilized to treat several different epithelial subtypes of human breast cancers.

Thus, 3D spheroid cultures appear to require oxidative mitochondrial metabolism, related to the re-use of monocarboxylic acids (ketones or L-lactate), for the proliferative anchorage-independent expansion of cancer stem cells.

### Functional effects of inhibition of the mitochondrial ATP synthase (complex V) on mammosphere formation

Finally, to further validate that mammosphere formation is strictly dependent on oxidative mitochondrial metabolism, we used a well-established investigational compound that potently inhibits the mitochondrial ATP synthase (complex V), namely oligomycin A. Importantly, five protein components of the mitochondrial ATP synthesis were highly up-regulated in MCF7 mammospheres (ATP5B, ATP5A1, ATP5F1, ATP5H, ATP5O).

**Figure 4:**
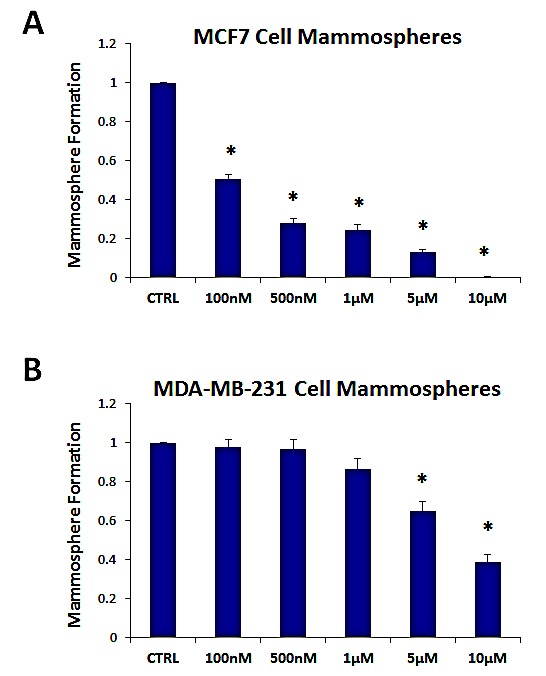
The mitochondrial ATP synthase inhibitor oligomycin A significantly reduces mammosphere formation in both MCF7 and MDA-MB-231 cells Note that oligomycin A effectively reduces mammosphere formation, with an IC-50 of ~100 nM in MCF7 cells (panel A) and ~5-10 μM in MDA-MB-231 cells (panel B). Thus, oligomycin A was nearly 50-100 times more potent in MCF7 cells. The vehicle-alone control was normalized to one. (*)p <3.2E-06.

Figure 4A, B shows the effects of increasing concentrations of oligomycin A on mammosphere formation. Note that oligomycin A significantly reduces mammosphere formation in MCF7 cells, with an IC-50 of ~ 100 nM. Oligomycin A also significantly inhibited mammosphere formation in MDA-MB-231 cells, but with less potency, with an IC-50 between 5-10 μM. As such, oligomycin A was 50-100 times less potent in MDA-MB-231 cells, as compared with MCF7 mammospheres.

Therefore, MCT1/2 inhibition may be a more effective strategy for eradicating cancer stem cells in multiple breast cancer types, rather than targeting the mitochondrial ATP synthase.

### Clinical relevance of mitochondrial targets in human breast cancers

To determine the potential clinical relevance of our findings, we next assessed whether the “metabolic targets” that we identified in mammospheres were also transcriptionally upregulated in human breast cancer cells *in vivo*.

For this purpose, we employed a published clinical data set of N=28 breast cancer patients in which their tumor samples were subjected to laser-capture micro-dissection, to physically separate epithelial cancer cells from their adjacent tumor stroma [17]. Table 3 presents a summary of these findings. Overall, 39 of the “metabolic targets” that we identified in mammospheres (Tables 1 & 2) were also transcriptionally elevated in human breast cancer cells *in vivo* (Table 3) and the majority of these targets were upregulated in both MCF7 and T47D mammospheres (21 out of 39, ~54%).

In light of these findings, the new “metabolic targets” that we identified in mammospheres are especially clinically relevant, for improving both the diagnosis and treatment of human breast cancers.

**Table 3:** “Metabolic Targets” Over-Expressed in Mammospheres are also Transcriptionally Up-regulated in Human Breast Cancer Cells In Vivo (Cancer Epithelia vs. Tumor Stroma)

Symbol	Gene Description	Fold-Upregulation (Epithelial/Stromal)	P-value
**CHCHD2**	Coiled-coil-helix-coiled-coil-helix domain-containing protein 2, mitochondrial	5.79	1.85E-07
**ACACA**	Acetyl-Coenzyme A carboxylase alpha	5.59	3.89E-07
**MCCC2**	Methylcrotonoyl-CoA carboxylase beta chain, mitochondrial	5.48	5.78E-07
**ATP5F1**	ATP synthase, H+ transporting, mitochondrial F0 complex, subunit B1	5.39	7.83E-07
ATP5O	ATP synthase subunit O, mitochondrial	5.12	2.13E-06
ATP5B	ATP synthase subunit beta, mitochondrial	5.04	2.75E-06
COX5B	Cytochrome c oxidase subunit 5B, mitochondrial	5.03	2.86E-06
ATP5A1	ATP synthase subunit alpha, mitochondrial	5.01	3.09E-06
PDHB	Pyruvate dehydrogenase E1 component subunit beta, mitochondrial	4.51	1.75E-05
**LRPPRC**	Leucine-rich PPR motif-containing protein, mitochondrial	4.34	3.15E-05
ECHS1	Enoyl-CoA hydratase, mitochondrial	4.05	8.22E-05
ATP5H	ATP synthase subunit d, mitochondrial	4.01	9.48E-05
**VDAC3**	Voltage-dependent anion-selective channel protein 3	3.94	1.19E-04
**HSPA9**	Stress-70 protein, mitochondrial	3.69	2.64E-04
**ATPIF1**	ATPase inhibitor, mitochondrial	3.60	3.48E-04
SLC25A5	Solute carrier family 25 (adenine nucleotide translocator), member 5	3.49	4.81E-04
**ACLY**	ATP Citrate Lyase, cytosolic	3.48	4.97E-04
**HSPD1**	60 kDa heat shock protein, mitochondrial	3.42	5.93E-04
TUFM	Elongation factor Tu, mitochondrial	3.38	6.74E-04
C21orf33	ES1 protein homolog, mitochondrial	3.31	8.40E-04
HADHA	Trifunctional enzyme subunit alpha, mitochondrial	3.27	9.34E-04
MRPS22	28S ribosomal protein S22, mitochondrial	3.27	9.31E-04
**HADH2**	Hydroxysteroid (17-Beta) Dehydrogenase 10; HSD17B10 protein	3.22	1.10E-03
PPA2	Inorganic pyrophosphatase 2, mitochondrial	3.19	1.17E-03
SQRDL	Sulfide:quinone oxidoreductase, mitochondrial	3.14	1.38E-03
**HADHB**	Mitochondrial trifunctional protein beta subunit	3.06	1.73E-03
**SUCLG2**	Succinyl-CoA ligase [GDP-forming] subunit beta, mitochondrial	3.03	1.89E-03
**PTCD3**	Pentatricopeptide repeat-containing protein 3, mitochondrial	2.98	2.15E-03
COX6B1	Cytochrome c oxidase subunit 6B1	2.97	2.21E-03
**MRPL17**	39S ribosomal protein L17, mitochondrial	2.94	2.38E-03
**LETM1**	LETM1 and EF-hand domain-containing protein 1, mitochondrial	2.81	3.45E-03
CCDC47	Coiled-coil domain-containing protein 47	2.70	4.68E-03
**DLAT**	Dihydrolipoyllysine acetyltransferase, pyruvate dehydrogenase complex	2.63	5.53E-03
**MCCC1**	Methylcrotonoyl-CoA carboxylase subunit alpha, mitochondrial	2.40	9.99E-03
AK2	Adenylate kinase 2, mitochondrial	2.20	1.59E-02
**ECH1**	Delta(3,5)-Delta(2,4)-dienoyl-CoA isomerase, mitochondrial	1.97	2.72E-02
**ACAT1**	Acetyl-CoA acetyltransferase, mitochondrial	1.93	2.97E-02
**BDH1**	D-beta-hydroxybutyrate dehydrogenase, mitochondrial	1.86	3.45E-02
ACO2	Aconitate hydratase, mitochondrial (EC 4.2.1.3)	1.83	3.64E-02

**-Transcriptional profiling data derived from the analysis of N=28 breast cancer patients are shown, high-lighting the levels of fold-upregulation observed in the epithelial cancer cell compartment (relative to the tumor stroma), and corresponding p-values derived from the analysis of these clinical samples.**

**-Proteins listed above (39 in total) were all upregulated either in MCF7 or T47D mammospheres (See Tables 1 & 2).**

**-Proteins shown in BOLD were commonly upregulated in both MCF7 and T47D mammospheres (21 out of 39 proteins).**

## DISCUSSION

Previous immuno-histochemical studies have shown that markers of cell proliferation (Ki67) and mitochondrial mass/function (TOMM20 and Complex IV activity) specifically co-localize to the basal stem cell layer in human oral mucosal tissue [18]. Interestingly, in this context, MCT1 was the most specific marker of the basal stem cell layer, suggesting that normal stem cells may use L-lactate and ketone bodies to fuel oxidative mitochondrial metabolism and stem cell proliferation [18]. Similarly, Ki67, mitochondrial markers and MCT1 also co-localized in aggressive head and neck tumor cells, consistent with the idea that amplification of mitochondrial metabolism may contribute to human tumor formation and cancer progression [18].

Numerous studies have also implicated ketone bodies and L-lactate metabolism in cancer biology and/or “stemness” in cancer cells. For example, treatment with mitochondrial fuels (such as L-lactate and 3-hydroxy-butyrate) is sufficient to stimulate mitochondrial biogenesis in MCF7 cells and dramatically increases the levels of gene transcripts normally expressed in embryonic, neuronal and hematopoietic stem cells [19]. Importantly, the transcriptional profiles of lactate- or ketone-treated MCF7 cells effectively predicted poor clinical outcome (tumor recurrence and metastasis) in ER-positive breast cancer patients [19]. Consistent with these findings, Cuyas et al. recently showed that cancer stem cells created by silencing of E-cadherin expression prefer to use L-lactate and ketone bodies as mitochondrial fuels [20].

Here, using unbiased label-free proteomics analysis, we show that mammospheres (a population of cells enriched in cancer stem cells and progenitor cells) functionally overexpress numerous mitochondrial proteins, related to mitochondrial biogenesis, electron transport, OXPHOS, ATP synthesis, as well as beta-oxidation and ketone re-utilization. The potential clinical relevance of these targets was further validated using a previously published data set of human breast cancer samples (N=28 patients), that were subjected to laser-capture microdissection, to separate the epithelial tumor cells from the adjacent tumor stroma [17]. Thus, these novel mitochondrial-based targets may reveal a metabolic “Achilles' Heel” to allow the eradication of cancer stem cells.

In accordance with this idea, we demonstrate that therapeutic targeting of MCT1/2 in cancer stem cells may be a viable strategy, via inhibiting the uptake of necessary key mitochondrial fuels (ketone bodies and L-lactate), that may be required for anchorage-independent growth, as well as cancer stem cell proliferative expansion and survival. Further validation was also provided by experiments with oligomycin A, a well-established inhibitor of the mitochondrial ATP synthase (complex V).

Interestingly, here we observed that the mitochondrial protein CHCHD2 was infinitely upregulated in both MCF7 and T47D mammospheres (Tables 1 and 2), and was also the most highly transcriptionally upregulated protein in the context of human breast cancer cells *in vivo* (Table 3). Thus, future studies may be warranted on the specific role of CHCHD2 in cancer stem cell metabolism. Currently, very little is known about CHCHD2. However, CHCHD2 has been previously implicated functionally in the response to hypoxia and in the transcriptional upregulation of members of the OXPHOS complexes, as well as a positive regulator of cell migration [21-23].

In conclusion, based on our quantitative proteomics analysis and functional validation studies using mammosphere cultures, we propose that mitochondria are new therapeutic targets for eradicating cancer stem cells, to prevent tumor recurrence, metastasis and poor clinical outcome in breast cancer patients.

## MATERIALS AND METHODS

### Materials

Breast cancer cell lines (MCF7, T47D and MDA-MB-231 cells) were purchased from the ATCC. AR-C155858 was obtained commercially from MedChem Express (UK). Gibco-brand cell culture media (DMEM/F12) was purchased from Life Technologies. Oligomycin A was obtained from Sigma-Aldrich.

### Mammosphere Culture

A single cell suspension was prepared using enzymatic (1x Trypsin-EDTA, Sigma Aldrich, #T3924), and manual disaggregation (25 gauge needle) to create a single cell suspension [5]. Cells were plated at a density of 500 cells/cm2 in mammosphere medium (DMEM-F12/B27/20ng/ml EGF/PenStrep) in non-adherent conditions, in culture dishes coated with (2-hydroxyethylmethacrylate) (poly-HEMA, Sigma, #P3932). Cells were grown for 5 days and maintained in a humidified incubator at 37°C at an atmospheric pressure in 5% (v/v) carbon dioxide/air. After 5 days for culture, spheres >50 μm were counted using an eye piece graticule, and the percentage of cells plated which formed spheres was calculated and is referred to as percentage mammosphere formation, and was normalized to one (1 = 100 %MSF). For proteomic analysis, mammospheres were collected by centrifugation at 800 rpm for 10 minutes.

### Label-free Quantitative Proteomics analysis

Cell lysates were prepared for trypsin digestion by sequential reduction of disulphide bonds with TCEP and alkylation with MMTS [24]. Then, the peptides were extracted and prepared for LC-MS/MS. All LC-MS/MS analyses were performed on an LTQ Orbitrap XL mass spectrometer (Thermo Scientific, San Jose, CA) coupled to an Ultimate 3000 RSLCnano system (Thermo Scientific, formerly Dionex, The Netherlands). Xcalibur raw data files acquired on the LTQ-Orbitrap XL were directly imported into Progenesis LCMS software (Waters Corp., Milford, MA, formerly Non-linear dynamics, Newcastle upon Tyne, UK) for peak detection and alignment. Data were analyzed using the Mascot search engine. Five replicates were analyzed for each sample type (N = 5). Statistical analyses were performed using ANOVA and only fold-changes in proteins with a p-value less than 0.05 were considered significant.

A more detailed proteomics protocol is provided as Supplementary Information.

### Data Mining

To firmly establish the clinical relevance of our results from the quantitative proteomics analysis of mammosheres, we re-analyzed the transcriptional profiles of epithelial breast cancer cells and adjacent tumor stromal cells that were physically separated by laser-capture microdissection (from N=28 human breast cancer patients) [17].

## SUPPLEMENTARY MATERIAL


