# Supplementary material for: Mitochondria as new therapeutic targets for eradicating cancer stem cells: Quantitative proteomics and functional validation via MCT1/2 inhibition
Source: Oncotarget. 2014 Nov 15;5(22):11029–37. doi: 10.18632/oncotarget.2789 (PMC4294326; doi:10.18632/oncotarget.2789)
Supplement: Supplementary file 1 [file oncotarget-05-11029-s001.pdf]

Mitochondria as new therapeutic targets for eradicating cancer stem cells: Quantitative proteomics and functional validation via MCT1/2 inhibition

Supplemental Information: Detailed Methods for Quantitative Label-free Proteomics.

Lysate reduction, alkylation and digestion.

5µgs of cell lysate was reduced with TCEP, alkylated with MMTS and digested with trypsin, as previously described in the iTRAQ protocol [1]. Digests were subsequently vacuum dried.

HILIC Solid Phase Extraction.

Dried digests were resuspended in 100µl 90% (v/v) acetonitrile, 5mM ammonium formate pH 2.7. PolyHYDROXYETHYL A SPE cartridges (PolyLC #SPEHY1203-SYR33 12µm, 300mg) were preconditioned in 2mls of 90% (v/v) acetonitrile, 5mM ammonium formate pH 2.7 followed by 2mls of water 5mM ammonium formate pH 2.7 followed by 6mls 90% (v/v) acetonitrile, 5mM ammonium formate pH 2.7. Sample was loaded onto the cartridge followed by washing with 10mls of 90% (v/v) acetonitrile, 5mM ammonium formate pH 2.7. Peptides were then eluted in 500ul 9% (v/v) acetonitrile, 5mM ammonium formate pH 2.7. Eluted peptides were then dried by vacuum centrifugation.

LCMS data acquisition.

500ng of peptides were separated utilizing an RSLCnano HPLC (Dionex) as detailed below. Each sample was loaded onto a Acclaim pepmap C18 trap column (300 µm internal diameter [ID], 5 µm 2 cm) in water, 1% (v/v) acetonitrile, and 0.05% (v/v) trifluoroacetic acid at a flow rate of 8 µl/min for 5 min. Peptides were then separated using an Acclaim pepmap C18 column (75um ID, 2 µm 50 cm) with a gradient of 1–35% (v/v) of acetonitrile, 0.1% formic acid over 45min at a flow rate of 300 nl/min. The nano liquid chromatography (nLC) effluent was sprayed directly into the LTQ-Orbitrap XL mass spectrometer aided by the Proxeon nano source at a voltage offset of 1.7 kV. The mass spectrometer was operated in parallel data-dependent mode where the MS survey scan was performed at a nominal resolution of 60,000 (at mass/charge [m/z] 400) in the Orbitrap analyzer in an m/z range of 400–2000. The top three precursors with charge states of 2, 3 and 4 were selected for collision induced dissociation (CID) in the LTQ at a normalized collision energy of 35%. MS2 gas phase fractionation was employed to reduce redundancy within the dataset. Each sample was analyzed 5 times, in each case MS1 data was collected in a m/z range of 400-2000 and MSMS acquisition was permitted only for ions of charge 2, 3 and 4 in the m/z range cuts of 400-526, 526-623, 623-727, 728-865 and 866-2000. These gas phase fraction windows were calculated empirically from a pooled pilot injection to ensure each m/z window contained equal numbers of ions with charges considered for MSMS and Dynamic exclusion was enabled to prevent the selection of a formally targeted ion for a total of 20 sec.

Reference

1. Holland M, Castro FV, Alexander S, Smith D, Liu J, Walker M, Bitton D, Mulryan K, Ashton G, Blaylock M, Bagley S, Connolly Y, Bridgeman J, Miller C, Krishnan S, Dempsey C, Masurekar A, Stern P, Whetton A, Saha V. 2011. RAC2, AEP, and ICAM1 expression are associated with CNS disease in a mouse model of pre-B childhood acute lymphoblastic leukemia. Blood. 118(3):638-49.
